# Comparison of ultracentrifugation and density gradient separation methods for isolating Tca8113 human tongue cancer cell line-derived exosomes

**DOI:** 10.3892/ol.2014.2373

**Published:** 2014-07-23

**Authors:** ZHUOYUAN ZHANG, CHENXING WANG, TANG LI, ZHE LIU, LONGJIANG LI

**Affiliations:** 1 State Key Laboratory of Oral Diseases, West China School of Stomatology, Sichuan University, Chengdu, Sichuan 610041, P.R. China; 2 Department of Head and Neck Cancer Surgery, West China School of Stomatology, Sichuan University, Chengdu, Sichuan 610041, P.R. China

**Keywords:** tongue squamous cell carcinoma, exosome, ultracentrifugation, density gradient separation

## Abstract

The aim of the present study was to compare the method of ultracentrifugation and density gradient separation for isolating Tca8113 human tongue squamous cell carcinoma cell line-derived exosomes. The exosomes were obtained from the culture supernatant of cultured Tca8113 cells, respectively, followed by identification with transmission electron microscopy observation and western blot analysis. The two different methods were then compared by the morphology, the distribution range of the particle size and the concentration of proteins of the extracted exosomes. *In vitro*, Tca8113 cells can secrete a large amount of vesicle-like structures, which are identified as exosomes by the presence of the surface markers, Hsp-70 and Alix. The protein profile of the two products are almost the same, however the particle size distribution of the exosomes extracted with density gradient centrifugation are more limited, between 40–120 nm, and these have a higher protein concentration. The results indicate that Tca8113 cells can secrete exosomes *in vitro*, and the density gradient separation methods for purifying exosomes is improved, which is helpful for future research and application of exosomes.

## Introduction

Exosomes are bioactive vesicles derived from the endosomal membrane system of the cell, followed by secretion into the surrounding body fluids, with diameters ranging from 40–100 nm and a density from 1.13–1.19 g/ml. Exosomes were officially named by Johnstone *et al* in 1987, who found that microvesicles can assist maturing erythrocytes to eliminate the transferring receptors as well as other dumped proteins (1). However, numerous studies have identified that exosomes can be produced by various types of cells, including different types of epithelial and nerve cells (2), and have even been detected in various body fluids (3–5). Exosomes are saucer-like vesicles under electron microscopy, which contain special proteins, lipids, RNA and micro-RNAs (6,7). They can be identified by Alix, Tsg101, heat shock protein 70 (Hsp70), and cluster of differentiation 63 (CD63), CD81 and CD9, and they play different roles in various pathological conditions. Thus far, in addition to participating in metabolizing products, it has been demonstrated that exosomes possess a number of biological functions, including immune regulation, communication of cells, matrix remodeling, signaling pathways activating through transferring growth factor or receptor, intercellular exchanging of oncoprotein and oncogene, and induction of angiogenesis and regulation of the treatment reaction. In particular, their significant role in intercellular communication has gained more and more attention in studies (1,8–11). Currently, there are three main methods for extraction of exosomes: i) Classic ultracentrifugation (12); ii) density gradient centrifugation, which is widely used at present (13); and iii) the technology of immunomagnetic capture (14–16). Due to the high cost and lack of specific antigens, the certain advantages of magnetic bead technology have resulted in its limited application. At present, the other two methods have a lack of a systematic comparison. The aim of the present study was to demonstrate that the Tca8113 human tongue squamous cell carcinoma cell line can secrete exosomes, and to perform an extensive evaluation of the methods widely used for extracting the exosomes, including density gradient centrifugation and ultracentrifugation.

## Materials and methods

### Cell culture and collection of required culture medium

The human tongue squamous cell carcinoma cell line, Tca8113, was obtained from the Cell Institute, Chinese Academy of Sciences (Shanghai, China) and cultured in Dulbecco’s modified Eagle’s medium (DMEM; Hyclone Laboratories, Inc., Logan, UT, USA) containing 10% fetal bovine serum (FBS; Gibco-BRL, Carlsbad, CA, USA) with 5% CO_2_ at 37°C, and cells were passaged when they were 90–100% confluent. Tca8113 cells were washed twice with 10 ml phosphate-buffered saline (PBS) and cultured for 48 h in 5 ml DMEM media with 10% FBS, which was previously centrifuged at 100,000 × g for 70 min to eliminate bovine-derived exosomes. Subsequently, ~50 ml culture medium (CM) was collected and stored at −20°C for later use.

### Ultracentrifugation exosome (UC-Exo) isolation

Exosomes were isolated from the required cell culture supernatant as described previously (12). Briefly, the cell culture supernatant was centrifuged (300 × g for 10 min; 2,000 × g for 20 min to eliminate dead cells; and 10,000 × g for 30 min to remove debris) and then pelleted by ultracentrifugation at 100,000 × g for 70 min at 4°C. The pellet was resuspended in 1 ml PBS and re-centrifuged (100,000 × g, 70 min), as aforementioned. The products (UC-Exo) were resuspended in 200 μl PBS and passed through 0.22-μm microcentrifuge filters (Sigma-Aldrich, St. Louis, MO, USA) prior to being stored at −80°C.

### Density gradient exosome (DG-Exo) isolation

Exosomes were isolated using a method described by Lamparski *et al* (17). Briefly, the collected CM was centrifuged at 2000 × g for 30 min to remove cellular debris. The processed supernatant was concentrated by centrifugation for 50 min at 1,000 × g in a 100 kDa molecular weight cut-off hollow-fibre membrane (Millipore, Bedford, MA, USA). The concentrated products were collected and added to an ultracentrifuge tube with a 30% sucrose/D_2_O cushion (density, 1.210 g/cm^3^) at the bottom of the tube, followed by ultracentrifugation (Sorvall Ultra Pro 80; Kendro Laboratory Products Ltd., Newtown, CT, USA) in a Surespin 630 swinging bucket (Thermo Fisher Scientific, Rockford, IL, USA) at 100,000 × g at 4°C for 70 min. Subsequently, the cushion was collected, followed by being washed and concentrated twice with PBS by centrifuging for 50 min at 1,000 × g in the aforementioned capsule. The following steps were consistent with the former method.

### Analysis of the laser particle size

The analysis of the particle size was carried out and repeated three times with a laser diffraction instrument (Malvern Zetasizer Nano ZS90; Malvern Instruments Ltd., Malvern, UK). A 30-μl aliquot of exosomes isolated respectively from the two types of methods was diluted with PBS to 1 ml and transferred to the specific tube subsequent to repeatedly blowing. The particle size was measured every 0.5 min for the duration of the test and the results were automatically recorded.

### Transmission electron microscopy (TEM)

The process of observation through TEM were performed as previously described (16) with slight alterations. Briefly, a 20 μl aliquot of exosome preparations were placed onto formvar-coated 200-mesh copper grids (ProSciTech, Queensland, Australia) for 1 min at room temperature and allowed to dry through filter paper. The grids were subsequently washed twice with water for 5 min and stained with 20 g/l uranyl acetate in water (ProSciTech) for 1 min. The grids were examined at an acceleration voltage of 100 kV using a JEOL JEM-2100 TEM (JEOL USA, Inc., Peabody, MA, USA).

### Protein extraction and concentration determination

As a result of the pre-experiment, the highest concentration of protein appeared in the certain instances when 100 μl lysis buffer was added to the same volume of samples. According to the total protein extraction kit instructions (Bi Yuntian Biological Technology Institution, Shanghai, China), 100 μl lysis buffer was added to the same volume of samples on ice, followed by shaking wildly on the horizontal shaking platform for 15 min and centrifuging at 15,558 × g at 4°C for another 15 min. Subsequently, the supernatant was obtained. Finally, a 20-μl aliquot of the supernatants was used for the determination of the protein concentration with the bicinchoninic acid (BCA) Protein Assay kit (Bi Yuntian Biological Technology Institution).

### SDS-page gel electrophoresis with Coomassie brilliant blue staining and western blotting

Total cellular proteins were loaded and run on 10% SDS gels (Beyotime Institute of Biotechnology, Shanghai, China) and then either transferred onto polyvinylidene fluoride membranes (Bio-Rad, Hercules, CA, USA) or stained with Coomassie brilliant blue (Beyotime Institute of Biotechnology). The membranes were blocked in 5% (w/v) skimmed milk in Tris-buffered saline with Tween 20 and incubated at 4°C with primary antibodies against monoclonal mouse anti-human Alix (1:1,000; Santa Cruz Biotechnology, Inc., Santa Cruz, CA, USA) or Hsp70 (1:1,000; Santa Cruz Biotechnology, Inc.) overnight. The polyclonal rabbit anti-goat IgG-horseradish peroxidase secondary antibodies (Wuhan Boster Biological Technology, Ltd., Wuhan, China) were incubated at room temperature for 1 h and the membranes were visualized by the Amersham ECL Select detection system (Amersham Pharmacia Biotech, Little Chalfont, UK).

### Statistical analysis

All data were performed using SPSS 13.0 (SPSS, Inc., Chicago, IL, USA) statistical software. A paired t-test was used to compare the protein concentration between the two methods. P<0.05 was considered to indicate a statistically significant difference.

## Results

### Particle size distribution of the exosomes

**Figure 1:**
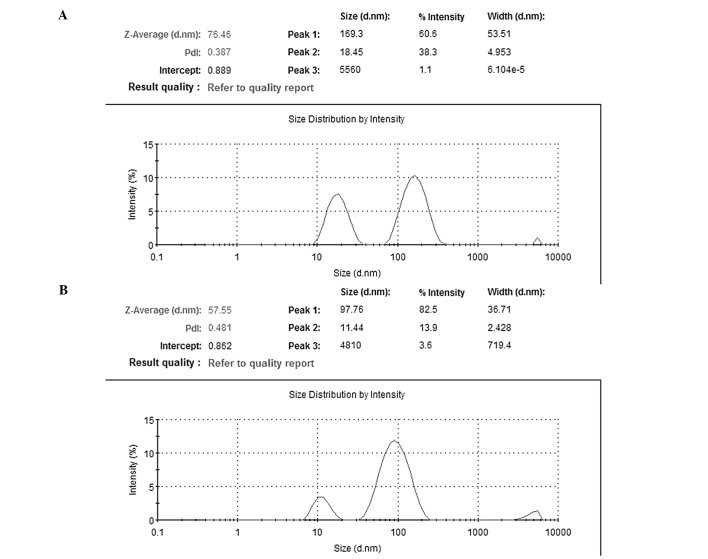
Particle size of exosomes isolated respectively from two kinds of methods were measured by a laser diffraction instrument. (A) The UC-Exo were widely distributed, >200 nm. (B) The diameter of DG-Exo mostly ranged uniformly from 30 to 120 nm. UC-Exo, ultracentrifugation exosome; DC-Exo, density gradient-Exo.

The particle size of the exosomes isolated respectively from the two types of method were measured by a laser diffraction instrument. The results revealed that the range of the particle size of UC-Exo (Fig. 1A) was wider than that of DG-Exo (Fig. 1B), even >200 nm, whilst the diameters of DG-Exo were mostly in a uniform range from 30–120 nm. The values of the polydispersity index were 0.387 and 0.481 for UC-Exo and DG-Exo, respectively, and were within the normal range.

### Morphology observation of the exosomes

**Figure 2:**
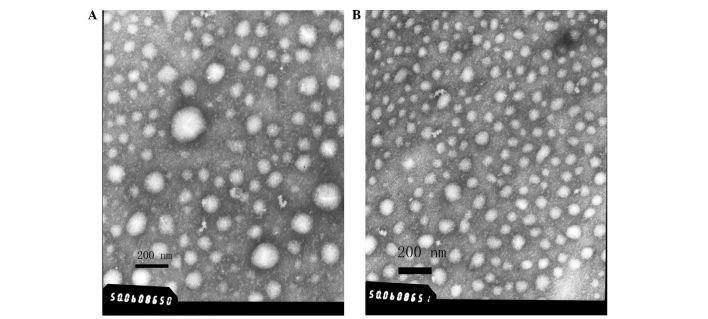
(A) UC-Exos and (B) DG-Exos were characterized by electron microscopy. The results showed nano-sized vesicles of a 30–150-nm diameter, with a ‘round-shaped’ morphology. Exosomes from each method were negatively stained with uranyl acetate and viewed by electron microscopy. The scale bar represents 200 nm (magnification, ×50,000). UC-Exo, ultracentrifugation exosome; DC-Exo, density gradient-Exo.

Morphological analysis of the UC-Exo and DG-Exo samples using TEM revealed the same results that the vesicles were comprising round-shaped 30–150-nm diameter vesicles, which is consistent with the aforementioned exosomes (Fig. 2), while the differences with the traditional cup-shape may be associated with the preparation of the samples. However, certain UC-Exo (Fig. 2A) samples contained vesicles that were >200 nm in diameter, which could even be suspected to be a class of apoptosis body, while the DG-Exo were well-distributed (Fig. 2B).

### Determination of the protein concentration of the exosomes

**Figure 3:**
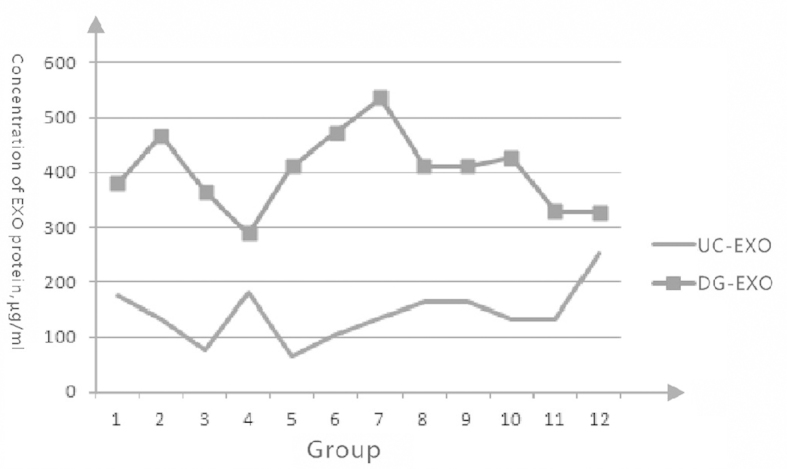
Results of a line graph derived from Table I. A paired t-test, P<0.05, implies that the difference was statistically significant.

The determination of the total protein concentration of the UC-Exo and DG-Exo samples were compared using the BCA method. The measurement included 12 separate samples from UC-Exo and DG-Exo (Table I) and the correlation coefficient was 0.99. Fig. 3 shows the curve comparison chart generated by Table I, with a paired t-test, P<0.05, which indicated that the difference had a statistical significance.

### Protein composition of the exosomes

**Figure 4:**
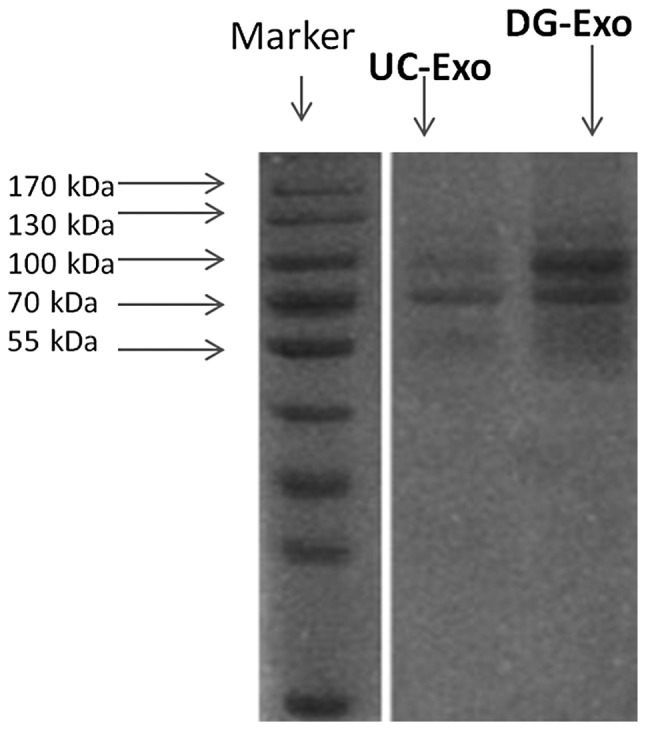
Protein composition of exosomes. The first longitudinal stripe on the right side was the marker, which was the indicator of molecular weight of the protein, while the other two longitudinal stripes represented the distribution of protein extracted from UC-Exo (left) and DG-Exo (right), respectively. The bands of the groups were similar, which contained the molecular weights of 55, 70, 100 and 130 kDa, while 170 kDa was not obvious. UC-Exo, ultracentrifugation exosome; DC-Exo, density gradient-Exo.

The proteins were separated by SDS-PAGE gel electrophoresis and stained by Coomassie brilliant blue. The results showed that the bands of the two samples were almost the same and were mainly distributed between 40–130 kDa with obvious separated strips (Fig. 4).

### Identification of the molecular markers

**Figure 5:**
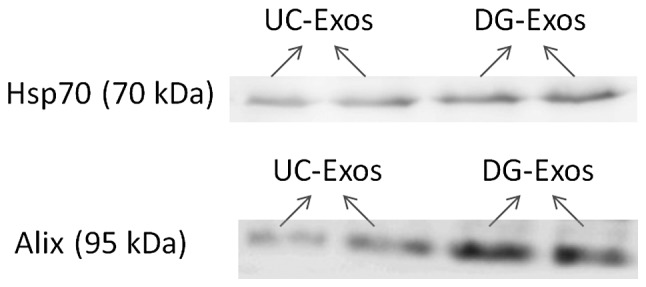
UC-Exos and DG-Exos were characterized by western blotting. For western blotting, each exosome preparation (20 μl) was separated by 1D-SDS-PAGE, followed by being transferred onto polyvinylidene fluoride membranes, and probed with exosome markers Alix and Hsp70. UC-Exo, ultracentrifugation exosome; DC-Exo, density gradient-Exo; Hsp70, heat shock protein 70.

The molecular phenotype of the exosomes was typical of exosomes from other sources. In the present study, it was found that Hsp70 and Alix, the protein markers of exosomes, were detected similarly in the purified exosomes with various methods (Fig. 5).

## Discussion

Exosomes may participate in cell communication by delivering proteins, RNA and miRNA (18–20) and has the ability to induce or suppress the immune system (21). Exosomes are capable of promoting angiogenesis, remodelling the microenvironment and promoting tumor growth (22,23). Their use in diagnosis and treatment have been confirmed (3,4,24). However, the current area of study offering the most promise lies with isolating and extracting high quality exosomes.

The significant reference standard to judge exosomes purification methods is based on the ability to remove other membranous particles and concentrate protein. Although ultracentrifugation is the most widely used method for exosomes isolation, it is limiting in these respects, so a more specific method is required. In the present study, two strategies were compared for purifying the human tongue cancer cell line Tca8113-derived exosomes; ultracentrifugation and density gradient separation. The efficacy of the two strategies was judged by TEM, particle size distribution and cursory proteome profiling of the enrichment of typical exosomal markers, including Alix and Hsp70, which was confirmed by western blot analysis and protein concentration.

Experimental results show that the exosomes extracted from the two methods, comprising round-shaped 30–150-nm diameter vesicles, are consistent with exosomes reported previously (16,25). However, the coexistence of the larger vesicles with UC-Exo is unknown and presumably not due to the vesicles being slightly clumped together or due to other mixed impurities. Another study has noted that steps, including freezing and thawing, or multiple centrifugal steps do not affect exosome size and shape (17). Western blot analysis revealed the presence of the exosome markers Alix and Hsp70 in both methods, which showed that both of the methods can extract the exosomes. In addition, the results of laser particle size measurement revealed that UC-Exo had a wider range of diameter distribution and a larger proportion of vesicles with diameters >200 nm, while the DG-Exo was more uniform and the majority of diameters were distributed between 30–150 nm with an average of 87.3 nm. This shows that the latter samples have a higher purity. Through SDS-PAGE gel electrophoresis and Coomassie brilliant blue staining, the bands of the two samples were found to be almost the same and mainly distributed between 40–130 kDa with obvious separated strips, while the 170 kDa was not obvious. However, by comparing the two methods with protein concentration and western blot semi-quantitative analysis, it was found that the total protein concentration of DG-Exo was higher and had a higher proportion of target protein, which indicated that density gradient centrifugation has a higher extraction efficiency. Additionally, the density gradient centrifugation used a 30% sucrose/heavy water cushion, which has a density distribution range that is consistent with exosomes and is a type of variable purification, and has been used widely to purify exosomes (26). The repeated purification with ultrafiltration also produces samples with a higher purity and improved quality. Therefore, density gradient centrifugation is more comprehensive and more efficient than ultracentrifugation in the extraction of exosomes. However, there are specific problems attached, including the higher cost, the higher requirements of equipment and technical ability, the time required is longer and the separation and extraction of heavy water at a high-quality is difficult. In addition, future studies could be conducted to identify whether the cell damage from heavy water can be simply eliminated by repeated dilution of the purification and whether this will effect the subsequent experiment, whether the material existing in the extraction of the sample whose diameter is >200 nm can be further purified or excluded and whether one or more specific markers and extraction methods for exosomes will be a problem that requires solving. In conclusion, both preparations contained vesicles with sizes of 30–150 nm and expression of the exosome markers, Alix and HSP70. However, density gradient separation was considered to be the efficient method to isolate exosomes, as it was able to enrich exosome markers, and exosome-associated proteins by at least two-fold more than the other methods studied. Protein, lipid, mRNA and microRNA analyses of highly-purified vesicles will lead to significant advances in exosome characterization, and facilitate a deeper understanding of their biological functions.

## Figures and Tables

**Table I:** Determination of protein concentration of exosomes purified by two different methods.

Sample	UC-Exo concentration, μg/ml	DG-Exo concentration, μg/ml
1	174.957	380.910
2	132.555	467.076
3	75.361	364.452
4	179.888	288.936
5	64.325	410.922
6	103.051	471.916
7	135.000	535.814
8	165.013	410.922
9	163.076	411.892
10	132.096	427.382
11	133.064	329.598
12	252.146	327.622

UC-Exo, ultracentrifugation exosome; DC-Exo, density gradient-Exo.
